# Supplementary material for: Short-term power load forecasting based on the CEEMDAN-TCN-ESN model
Source: PLoS One. 2023 Oct 26;18(10):e0284604. doi: 10.1371/journal.pone.0284604 (PMC10602357; doi:10.1371/journal.pone.0284604)
Supplement: Attachment — Submitted filename: Response to the comments20230216.docx [file pone.0284604.s001.docx]

**Dear editors and reviewers:**

Thank you for your letter and for the reviewer's comments concerning our manuscript entitled “Short-Term Power Load Forecasting Based on the CEEMDAN-TCN-ESN Model” (PLOS ONE, PONE-D-23-03222). These comments are all valuable and very helpful for revising and improving our paper and have important guiding significance to our research. We have studied the comments carefully and have made corrections that we hope will meet with approval. The modified manuscript has been improved and polished by professional services. All modifications have been marked in RED. The main corrections in the paper and the responses to the reviewers’ comments are as follows.

**Editor:**

**Comment 1:**

2. Our internal editors have looked over your manuscript and determined that it is within the scope of our Smart Energy Systems Call for Papers. The Collection will encompass the latest research in smart grid technologies, including information technologies, device integration, distribution methods, and data mining, all towards improving the efficiency of energy supply networks. Additional information can be found on our announcement page: https://collections.plos.org/call-for-papers/smart-energy-systems/. If you would like your manuscript to be considered for this collection, please let us know in your cover letter and we will ensure that your paper is treated as if you were responding to this call. If you would prefer to remove your manuscript from collection consideration, please specify this in the cover letter.

**Response:**

Thank you for your comments. I would like our manuscript to be considered for this collection.

**Comment 2:**

4. We note that Figure 4 in your submission contain map images which may be copyrighted. All PLOS content is published under the Creative Commons Attribution License (CC BY 4.0), which means that the manuscript, images, and Supporting Information files will be freely available online, and any third party is permitted to access, download, copy, distribute, and use these materials in any way, even commercially, with proper attribution. For these reasons, we cannot publish previously copyrighted maps or satellite images created using proprietary data, such as Google software (Google Maps, Street View, and Earth). For more information, see our copyright guidelines: http://journals.plos.org/plosone/s/licenses-and-copyright.

We require you to either (1) present written permission from the copyright holder to publish these figures specifically under the CC BY 4.0 license, or (2) remove the figures from your submission:

a. You may seek permission from the original copyright holder of Figure 4 to publish the content specifically under the CC BY 4.0 license.

We recommend that you contact the original copyright holder with the Content Permission Form (http://journals.plos.org/plosone/s/file?id=7c09/content-permission-form.pdf) and the following text:

“I request permission for the open-access journal PLOS ONE to publish XXX under the Creative Commons Attribution License (CCAL) CC BY 4.0 (http://creativecommons.org/licenses/by/4.0/). Please be aware that this license allows unrestricted use and distribution, even commercially, by third parties. Please reply and provide explicit written permission to publish XXX under a CC BY license and complete the attached form.”

Please upload the completed Content Permission Form or other proof of granted permissions as an "Other" file with your submission.

In the figure caption of the copyrighted figure, please include the following text: “Reprinted from [ref] under a CC BY license, with permission from [name of publisher], original copyright [original copyright year].”

b. If you are unable to obtain permission from the original copyright holder to publish these figures under the CC BY 4.0 license or if the copyright holder’s requirements are incompatible with the CC BY 4.0 license, please either i) remove the figure or ii) supply a replacement figure that complies with the CC BY 4.0 license. Please check copyright information on all replacement figures and update the figure caption with source information. If applicable, please specify in the figure caption text when a figure is similar but not identical to the original image and is therefore for illustrative purposes only.

The following resources for replacing copyrighted map figures may be helpful:

USGS National Map Viewer (public domain): http://viewer.nationalmap.gov/viewer/

The Gateway to Astronaut Photography of Earth (public domain): http://eol.jsc.nasa.gov/sseop/clickmap/

Maps at the CIA (public domain): https://www.cia.gov/library/publications/the-world-factbook/index.html and https://www.cia.gov/library/publications/cia-maps-publications/index.html

NASA Earth Observatory (public domain): http://earthobservatory.nasa.gov/

Landsat: http://landsat.visibleearth.nasa.gov/

USGS EROS (Earth Resources Observatory and Science (EROS) Center) (public domain): http://eros.usgs.gov/#

Natural Earth (public domain): http://www.naturalearthdata.com/

**Response:**

Thank you for your reminder. We realize that this is an important issue. We have changed the map in Figure 4 to avoid potential copyright issues.

Figure 4. Research framework

**Reviewer #1:**

In this study, a hybrid CEEMDAN-TCN-ESN forecasting model is proposed for short-term load forecasting research. The works done in the paper are good, but to make it acceptable, some improvements must be made.

**Comment 1:**

1. Please check Eqs. (13)-(15), What is in the brackets?

**Response:**

Thank you for your comments.

We realize that it was unnecessary to note the dimension at the end of the formula, so we have removed the superfluous part in Eqs. (13)-(15).

**Comment 2:**

2. Some raw data should be displayed. The statistical results of raw data should be presented.

**Response:**

Thank you for your comments.

We have added some descriptive statistics of the original data to the article, including the name of the variable, the sample size, the mean, the standard deviation, the maximum and the minimum. The statistical results of the raw data are shown in Table 1.

Table 1: The statistical results of raw data

| Variable name | Sample size | Mean value | Standard deviation | Minimum value | Maximum Value |
| --- | --- | --- | --- | --- | --- |
| nat_demand | 17520 | 1224.629 | 188.256 | 85.193 | 1721.516 |

**Comment 3:**

3. MAPE can be added to show the performance.

**Response:**

Thank you for your comments.

Regarding this question, after some simple experiments, we found that the addition of MAPE has no effect on the selection of the model in this experiment. However, if MAPE is to be added, all experimental indicators in this paper will be slightly changed, and specific experimental indicators are difficult to completely reproduce. Therefore, it is necessary to reconduct the experiment and change the corresponding statistical charts of the evaluation indicators, which will increase the workload. Taking these into account, we decided to retain the original evaluation indicators.

**Comment 4:**

4. As written, some imprecision in word choice, grammar, and punctuation from the conveyance of the research.

**Response:**

Thank you for your comments. We carefully checked the article for punctuation, word choice and grammar and further corrected the language problems.

**Comment 5:**

5. The literature review should be improved since many advanced models (machine learning, deep learning, and so on) have been proposed in recent years. Their merits and demerits should be discussed to enrich this study. Some of the examples are:

[1] https://doi.org/10.1016/j.energy.2021.121756

[2] https://doi.org/10.1016/j.dsm.2021.12.002

[3] https://doi.org/10.1016/j.dsm.2022.09.001

**Response:**

Thank you for your comments. In recent years, few scholars have used separate linear regression and time series models to forecast short-term power loads, so we did not delete the old references of these two parts. For relevant references on machine learning and deep learning, we deleted those earlier than 2020 and replaced them with newer and more advanced ones.

Matheus [8] proposed a hybrid learning model based on a dual decomposition approach. The locally weighted regression (STL) is used to decompose the original time series into seasonal, trend, and residual components. Then, variational mode decomposition (VMD) is adopted to decompose the STL residual into different frequencies, and seasonal naïve is used to handle the seasonal patterns. Moreover, in consideration of the nonlinearities of the remaining components, extreme learning machines, ridge regression, and support vector regression models are employed to handle the STL trend and VMD components.

Zhou et al. [9] proposed a novel predictive system based on a data denoising strategy, statistical predictive systems, artificial intelligence forecasting systems and a multiobjective optimization strategy. After the data denoising process, the reconstructed data are used as the input of different subsystems. Then, to obtain stable forecasting results, a multiobjective dragonfly algorithm is used to estimate the weight coefficient of the subsystems.

Lu et al. [13] proposed a long short-term memory-based model for energy consumption forecasting. The empirical wavelet transform (EWT) technology was first used to decompose the energy consumption sequence into several components. Then, each component is concatenated as the input of the attention-based LSTM model to obtain the predicted value of each component. Finally, the predicted value of each component is integrated to obtain the final output.

*[8] Ribeiro M H D M, da Silva R G, Ribeiro G T, et al. Cooperative ensemble learning model improves electric short-term load forecasting[J]. Chaos, Solitons & Fractals, 2023, 166: 112982.*

*[9] Zhou Y, Wang J, Lu H, et al. Short-term wind power prediction optimized by multi-objective dragonfly algorithm based on variational mode decomposition[J]. Chaos, Solitons & Fractals, 2022, 157: 111982.*

*[13] Peng L, Wang L, Xia D, et al. Effective energy consumption forecasting using empirical wavelet transform and long short-term memory[J]. Energy, 2022, 238: 121756.*

**Reviewer #2:**

The topic is important. However, the following problems must be handled.

**Comment 1:**

(a) How can the related parameters of the proposed CEEMDAN-TCN-ESN be set for better performance?

**Response:**

Thank you for your comments.

In this paper, the hyperparameters of the model used are basically selected default values. Usually, for data sets with a large amount of data, we will pay more attention to the parameter adjustment of the model to make the model achieve a better prediction effect. The data set used in this paper only contains less than 20,000 data points, so we focus on the selection of the model and the combination pattern of the algorithm and only perform some simple processing for the parameter adjustment of the model. For example, the number of “Max epochs” of the TCN model is determined according to the curve diagram of the change in the loss function with the number of epochs of the model. For the key hyperparameter values in the model, such as the “convolution kernel size” in the TCN model, the common 2 and 3 are selected and compared, and the number of “Nb filters” is selected and compared between 16, 32, 64 and 128. We know that the parameter adjustment process is of great significance to the deep learning model, so in the following research work, we will further explore the parameter setting of the hybrid model proposed in this paper.

**Comment 2:**

(b)The Conclusions have not been written well. It is better to explain briefly the research gaps, research objectives, and methodology, and then clearly present the main results obtained in the work.

**Response:**

Thank you for your comments. We have rewritten the Conclusions, tried to briefly explain the research gaps, research objectives, and methodology, and then clearly presented the main results obtained in the work.

Due to the complex characteristics of the data and many related influencing factors, this paper establishes a hybrid model of the TCN and ESN based on CEEMDAN decomposition and high- and low-frequency series reconstruction modes for the STLF problem. In this paper, CEEMDAN decomposition is used to decompose the original power load series into subseries with different frequency characteristics, and all the subseries are reconstructed according to their frequency characteristics to obtain the higher-frequency and the lower-frequency-trend series. Then, the same eight models are used to predict the higher-frequency and lower-frequency-trend series, and the best prediction model for the two reconstructed series are TCN and ESN, respectively. Finally, the prediction results for the two series are combined to output the final power load forecast results.

Through the experimental analysis and the comparative analysis of different models, the results show that (1) For the prediction of the original electricity load series, TCN has the best performance among the single prediction models chosen for this experiment, indicating that it can extract the features of the original time series to a large extent. BILSTM ranks second, and ARIMA is the worst, indicating that deep learning approaches generally outperform traditional statistical models in STLF. (2) CEEMDAN decomposition can fully extract time series features and significantly improve the prediction accruracy of the model. (3) Sequence reconstruction can fuse the sequences with similar frequency features in the subseries obtained from CEEMDAN decomposition to obtain two reconstructed series. In addition, the optimal model is selected for each of the two series to produce a hybrid model to further improve the prediction effect of the model. (4) The RMSE, MAE, and $R^{2}$ of the proposed hybrid model are 15.081, 10.944, and 0.994, respectively, which are higher than those of the other models. Compared to the best single prediction model (TCN), the RMSE is reduced by 51.3%, the MAE is reduced by 52.85%, and the $R^{2}$ is improved by 2.16%. Compared to the second-best model (CEEMDAN-TCN), the RMSE is reduced by 9.52%, the MAE is reduced by 17.39%, and the $R^{2}$ is improved by 0.20%.

The CEEMDAN-TCN-ESN model proposed in this paper performs well in STLF and has certain application prospects, but the model still has the following problems: The expenditure of the model is large, the depth of the TCN is shallow, and only the hour-by-hour forecasting of short-term power load data is performed. In the future, if the experimental budget is sufficient, deepening the depth of the network to explore the prediction accuracy of the proposed model for 3 h, 6 h, 12 h or even 24 h in the future and expanding the scope of the model could be considered.

**Comment 3:**

(c)Section 1.4: Some advances of deep learning methods with necessary analysis can be included to improve the importance (Multivariate wind speed forecasting based on multi-objective feature selection approach and hybrid deep learning model; Deep learning combined wind speed forecasting with hybrid time series decomposition and multi-objective parameter optimization; etc.).

**Response:**

Thank you for your comments.

In this paper, we add the application of deep learning in the prediction of wind speed, temperature and other fields to highlight its superiority in the prediction of time series in the field of natural science.

In recent years, deep learning models have been increasingly adopted in the research of time series such as wind speed and temperature. Compared with traditional statistical models, deep learning models often have better performance in the prediction task of multifeature complex time series. Similarly, deep learning is very important in the prediction task of short-term power loads.

**Comment 4:**

(d)Reference: Some old papers should be deleted. Please add new papers since 2021.

**Response:**

Thank you for your comments.

In recent years, few scholars have used separate linear regression and time series models to forecast short-term power loads, so we did not delete the old references of these two parts. For relevant references on machine learning and deep learning, we deleted those earlier than 2020 and replaced them with newer ones.

Matheus [8] proposed a hybrid learning model based on a dual decomposition approach. The locally weighted regression (STL) is used to decompose the original time series into seasonal, trend, and residual components. Then, variational mode decomposition (VMD) is adopted to decompose the STL residual into different frequencies, and seasonal naïve is used to handle the seasonal patterns. Moreover, in consideration of the nonlinearities of the remaining components, extreme learning machines, ridge regression, and support vector regression models are employed to handle the STL trend and VMD components.

Zhou et al. [9] proposed a novel predictive system based on a data denoising strategy, statistical predictive systems, artificial intelligence forecasting systems and a multiobjective optimization strategy. After the data denoising process, the reconstructed data are used as the input of different subsystems. Then, to obtain stable forecasting results, a multiobjective dragonfly algorithm is used to estimate the weight coefficient of the subsystems.

Lu et al. [13] proposed a long short-term memory-based model for energy consumption forecasting. The empirical wavelet transform (EWT) technology was first used to decompose the energy consumption sequence into several components. Then, each component is concatenated as the input of the attention-based LSTM model to obtain the predicted value of each component. Finally, the predicted value of each component is integrated to obtain the final output.

*[8] Ribeiro M H D M, da Silva R G, Ribeiro G T, et al. Cooperative ensemble learning model improves electric short-term load forecasting[J]. Chaos, Solitons & Fractals, 2023, 166: 112982.*

*[9] Zhou Y, Wang J, Lu H, et al. Short-term wind power prediction optimized by multi-objective dragonfly algorithm based on variational mode decomposition[J]. Chaos, Solitons & Fractals, 2022, 157: 111982.*

*[13] Peng L, Wang L, Xia D, et al. Effective energy consumption forecasting using empirical wavelet transform and long short-term memory[J]. Energy, 2022, 238: 121756.*

**Comment 5:**

(e) Please further highlight the contribution of this study.

**Response:**

Thank you for your comments. We further highlight the contribution of this study as follows.

In this study, a special combination model is designed by combining the modal decomposition technique and the dual prediction model. Compared with the research methods without time series decomposition or using only a single prediction model, it has higher prediction efficiency and accuracy. The main contributions of this paper are as follows.

(1) A special combination model is designed by combining the modal decomposition technique and the dual prediction model. Compared with the research methods without decomposition techniques or using only a single prediction model, it has higher prediction efficiency and accuracy.

(2) This paper is not limited to a single traditional statistical perspective or a single machine learning perspective but considers the prediction model from two fields at the same time. The respective classical models are selected as experimental alternatives to discover the fitting effects of different classes of models on data with different characteristics. By observing their respective performances in the original series, higher-frequency, and lower-frequency-trend series to achieve a two-way comparison between horizontal and vertical.

(3) A unique signal decomposition technique (CEEMDAN) is selected. Gaussian white noise is introduced to cover the white noise of the original sequence; therefore, the mode aliasing problem is solved. In addition, the reconstructed data are used for model prediction, which improves the prediction performance.

(4) In this study, four sets of experiments were conducted. The experiments include the prediction of a single model without the decomposition technique, a single prediction model with the decomposition technique but without sequence reconstruction, a single prediction model with the decomposition technique and sequence reconstruction, and a dual prediction model with the decomposition technique and sequence reconstruction.

(5) Three compact and rational indicators are selected as the evaluation metrics for evaluating whether the predictive result of the designed model is good. which includes the root mean square error, mean square error and $R^{2}$.

(6) A robustness analysis experiment was carried out, and the four experiments above were repeated on another data set. The experimental results show that the proposed hybrid prediction model has a good generalization ability.

Thank you and best regards.

Xuchu Jiang

[xuchujiang@zuel.edu.cn](mailto:xuchujiang@zuel.edu.cn)

School of Statistics and Mathematics

Zhongnan University of Economics and Law
