# Supplementary material for: Short-term power load forecasting based on the CEEMDAN-TCN-ESN model
Source: PLoS One. 2023 Oct 26;18(10):e0284604. doi: 10.1371/journal.pone.0284604 (PMC10602357; doi:10.1371/journal.pone.0284604)
Supplement: Attachment — Submitted filename: Response to the comments20230325.docx [file pone.0284604.s002.docx]

**Dear editors and reviewers:**

Thank you for your letter and for the reviewer's comments concerning our manuscript entitled “Short-Term Power Load Forecasting Based on the CEEMDAN-TCN-ESN Model” (PLOS ONE, PONE-D-23-03222R1). These comments are all valuable and very helpful for revising and improving our paper and have important guiding significance to our research. We have studied the comments carefully and have made corrections that we hope will meet with approval. The modified manuscript has been improved and polished by professional services. All modifications have been marked in RED. The main corrections in the paper and the responses to the reviewers’ comments are as follows.

**Reviewer #2:**

**Comment 1:**

This revision is improved. For the technology aspect, the contribution is not very strong. However, some of my advices such as the possible applications of deep learning methods in this field are not handled. The authors just discussed it in the response letter. It is better to analyzed in the further research with some references. The writing should be improved.

The contributions are given. But some are not convincing. 3-4 Points are enough.

**Response:**

Thank you for your comments. Combined with some references, we have further supplemented the application of deep learning methods in this field. In future studies, we would like to pay more attention to the parameter setting of algorithms and models in deep learning methods, and try to apply optimization algorithms to obtain better parameter values for model prediction, so as to improve the performance of the model. Finally, as for the contribution of the article, we have deleted the unconvincing part and kept four points in the article.

Lv et al. [9] proposed an effective hybrid model for wind speed forecasting tasks based on an improved hybrid time series decomposition strategy (HTD) and the novel multi-objective binary backtracking search algorithm (MOBBSA). Furthermore, the advanced Sequence-to-Sequence (Seq2Seq) predictor is used to obtain the final forecasting result. In addition, Lv et al. [10] also proposed the FWNSDEC-SSA-ConvLSTM model for enhancing the short-term wind speed forecasting performance. The filter-wrapper non-dominated sorting differential evolution algorithm incorporating K-medoid clustering (FWNSDEC) is designed to select key meteorological factors, and singular spectrum analysis (SSA) is used to decompose the meteorological factors. Then, the convolutional long short-term memory (ConvLSTM) network is adopted to obtain the final forecasting result.

*[9]Lv S X, Wang L. Deep learning combined wind speed forecasting with hybrid time series decomposition and multi-objective parameter optimization[J]. Applied Energy, 2022, 311: 118674.*

*[10]Lv S X, Wang L. Multivariate wind speed forecasting based on multi-objective feature selection approach and hybrid deep learning model[J]. Energy, 2023, 263: 126100.*

The main contributions of this paper are as follows.

(1) A special combination model is designed by combining the modal decomposition technique and the dual prediction model. Compared with the research methods without decomposition techniques or using only a single prediction model, it has higher prediction efficiency and accuracy.

(2) This paper is not limited to a single traditional statistical perspective or a single machine learning perspective but considers the prediction model from two fields at the same time. The respective classical models are selected as experimental alternatives to discover the fitting effects of different classes of models on data with different characteristics. By observing their respective performances in the original series, higher-frequency, and lower-frequency-trend series to achieve a two-way comparison between horizontal and vertical.

(3) In this study, four sets of experiments were conducted. The experiments include the prediction of a single model without the decomposition technique, a single prediction model with the decomposition technique but without sequence reconstruction, a single prediction model with the decomposition technique and sequence reconstruction, and a dual prediction model with the decomposition technique and sequence reconstruction.

(4) A robustness analysis experiment was carried out, and the four experiments above were repeated on another dataset. The experimental results show that the proposed hybrid prediction model has a good generalization ability.

Thank you and best regards.

Xuchu Jiang

[xuchujiang@zuel.edu.cn](mailto:xuchujiang@zuel.edu.cn)

Zhongnan University of Economics and Law
